# Laparoscopic Partial Nephrectomy for Multiple Complex Renal Masses in a Patient With Von Hippel-Lindau Syndrome: A Case Report

**DOI:** 10.7759/cureus.76761

**Published:** 2025-01-01

**Authors:** Hisham M Ghabbani, Rashad M Nassar, Faisal A Alsaleh, Abdullatif M Alhassan, Abdullah M Almousa, Abdulaziz A Albalawi, Hamad S Al-Akrash, Ahmed Y Alzahrani

**Affiliations:** 1 Urology, Prince Sultan Military Medical City, Riyadh, SAU; 2 Urology, King Khalid University Hospital, Riyadh, SAU; 3 Urology, Security Forces Hospital, Riyadh, SAU

**Keywords:** laparoscopic urology, urologic cancer, uro oncology, von hippel-lindau disease (vhl), von hippel-lindau syndrome (vhl)

## Abstract

Von Hippel-Lindau (VHL) syndrome, an autosomal dominant hereditary disorder, is characterized by the formation of multiple and often bilateral renal tumors alongside various other systemic manifestations. The management of renal masses in VHL patients poses a notable challenge due to the threat of tumor recurrence and the need to safeguard renal function. Over the years, advancements in surgical methods have transformed the treatment measures for renal masses in VHL patients. Among these advancements, laparoscopic partial nephrectomy has emerged as a pivotal cornerstone in the management of complex renal masses.

## Introduction

Von Hippel-Lindau (VHL) syndrome, an autosomal dominant hereditary disorder, is characterized by the formation of multiple and often bilateral renal tumors alongside various other systemic manifestations. The management of renal masses in VHL patients poses a notable challenge due to the threat of tumor recurrence and the need to safeguard renal function [1]. These patients may exhibit synchronous bilateral, multifocal tumors in the kidneys at an early age and often experience recurrence or new tumor formation even after aggressive partial nephrectomy [2]. In an effort to address this issue, minimize the surgical complications associated with multiple renal interventions, and reduce the risk of metastatic disease, a specific approach was proposed for such a population, advocating intervention with partial nephrectomy when the largest tumor reaches 3 cm [3].

Over the years, advancements in surgical methods have transformed the treatment measures for renal masses in VHL patients. Among these advancements, laparoscopic partial nephrectomy has emerged as a pivotal cornerstone in the management of multiple complex renal masses.

## Case presentation

A 32-year-old male who is medically and surgically free, with a family history of VHL, was referred to us as an incidental finding of multiple renal masses for investigation. Upon history, the patient is clinically unremarkable. The physical examination showed no findings. Complete blood count and complete metabolic profile were both insignificant.

**Figure 1:**
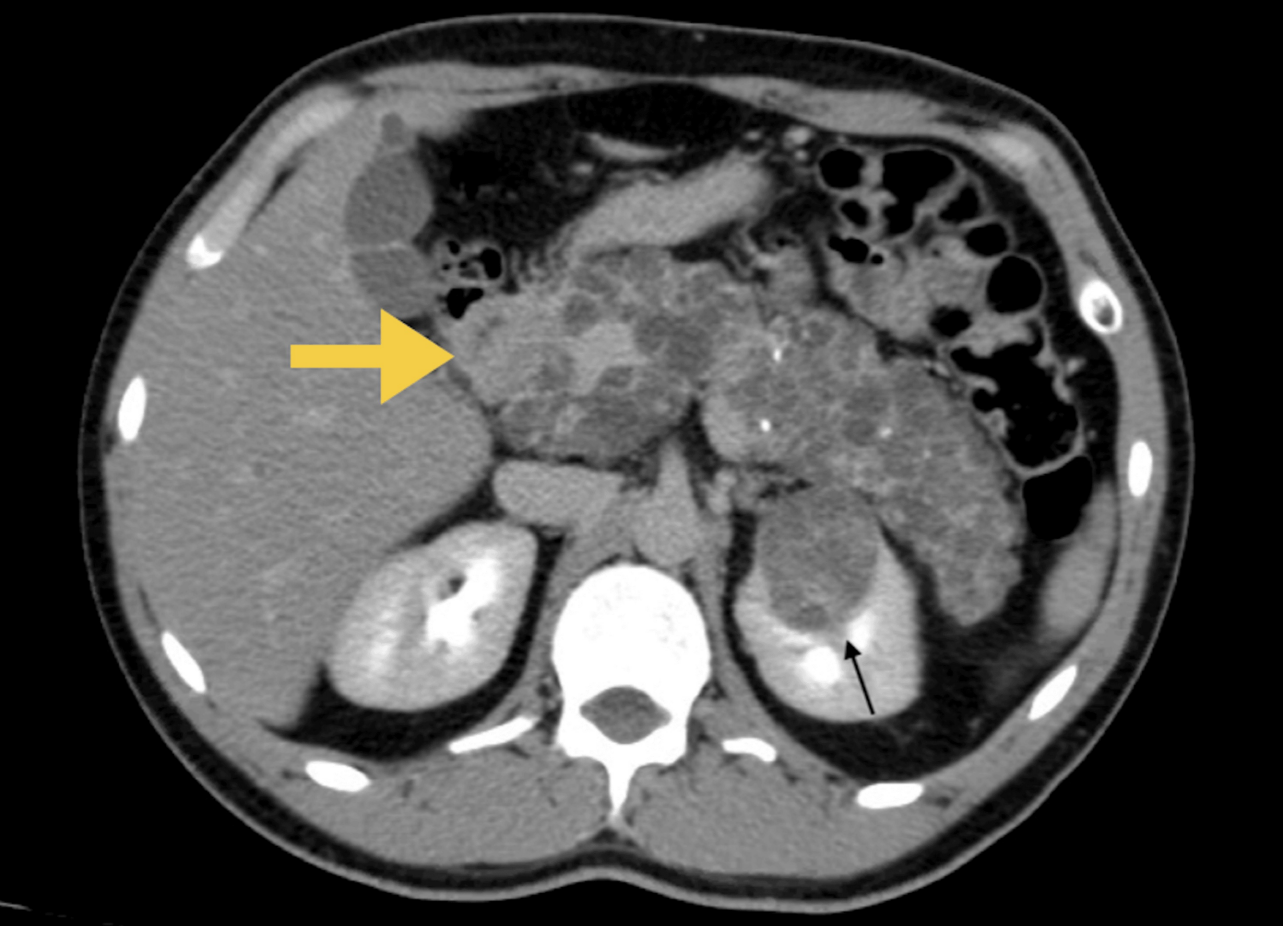
CT urography showing three complexes of multifocal RCC masses and pancreatic cyst/serous adenoma (axial view) CT: computed tomography, RCC: renal cell carcinoma

**Figure 2:**
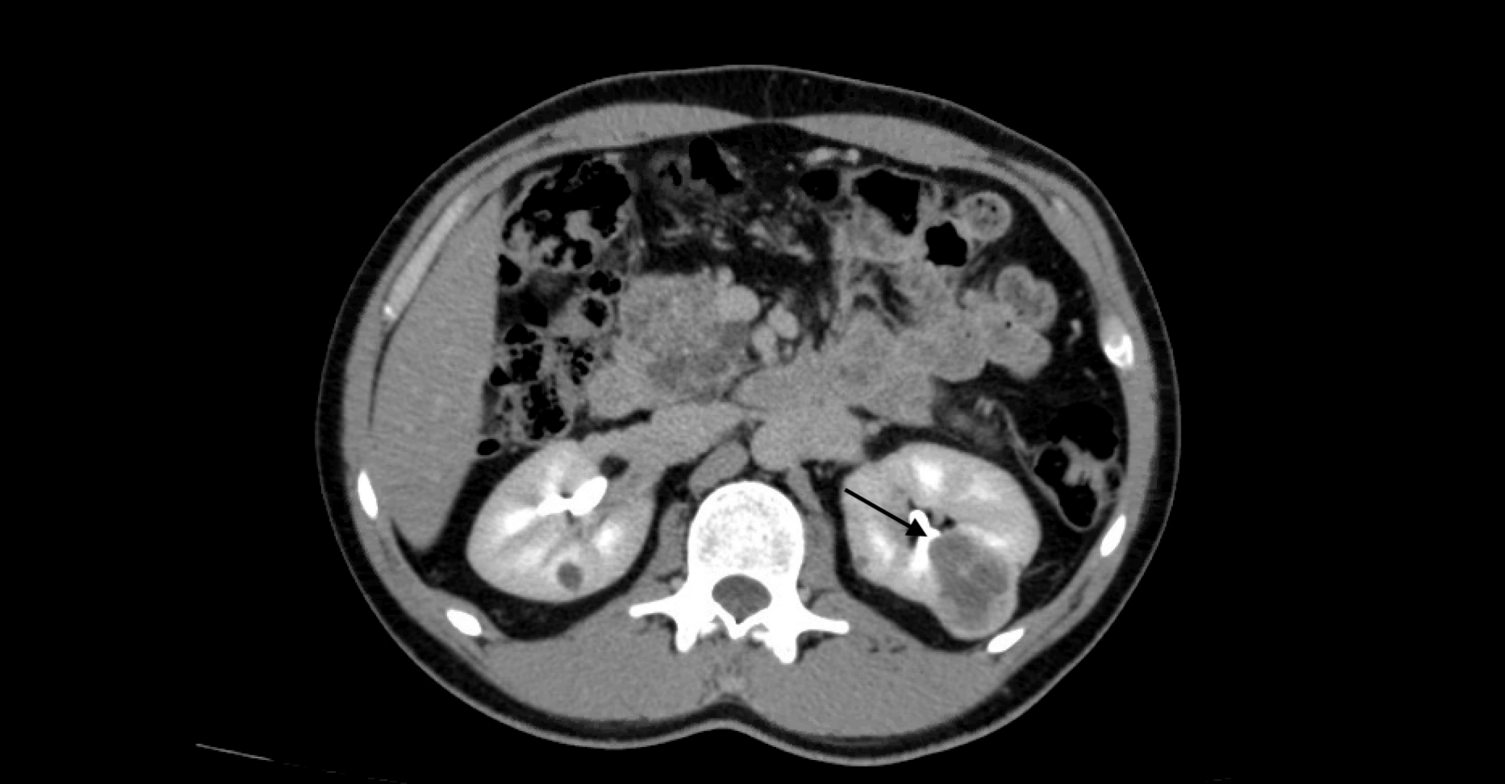
CT urography showing three complexes of multifocal RCC masses (axial view) CT: computed tomography, RCC: renal cell carcinoma

**Figure 3:**
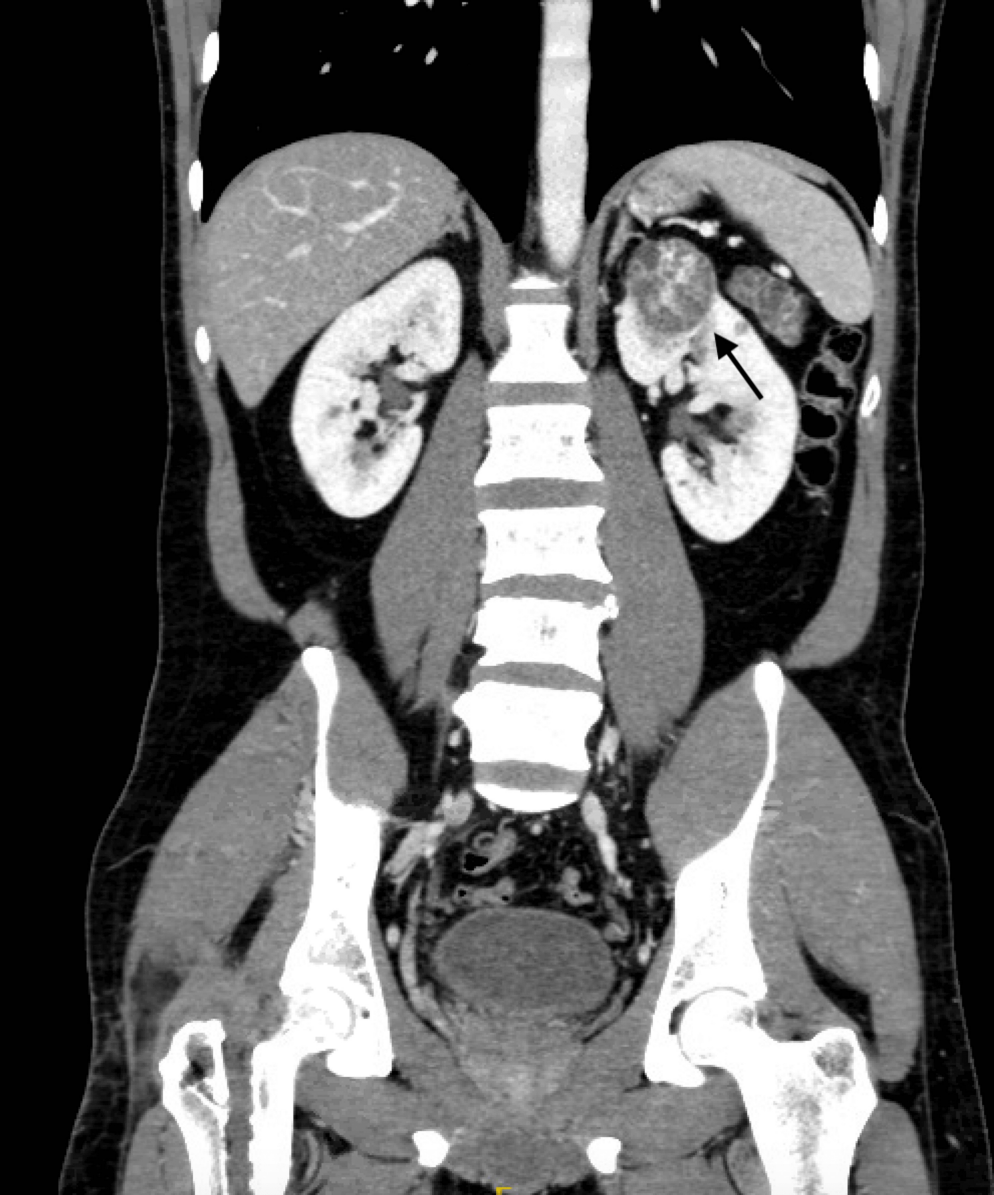
CT urography showing three complexes of multifocal RCC masses (coronal view) CT: computed tomography, RCC: renal cell carcinoma

A CT urography revealed three complex multifocal renal cell carcinoma (RCC) masses, the first arising from the anterior upper region measuring 3.5 cm, while the other two masses were arising from the posterior interpolar region, measuring 3.6 cm, 1.8 cm, and an entire replaced pancreatic mass lesion with pancreatic cyst/serous cystadenoma (Figures 1-3). There was no finding of venous tumor thrombus formation, lymph node metastases, or distant metastases.

**Figure 4:**
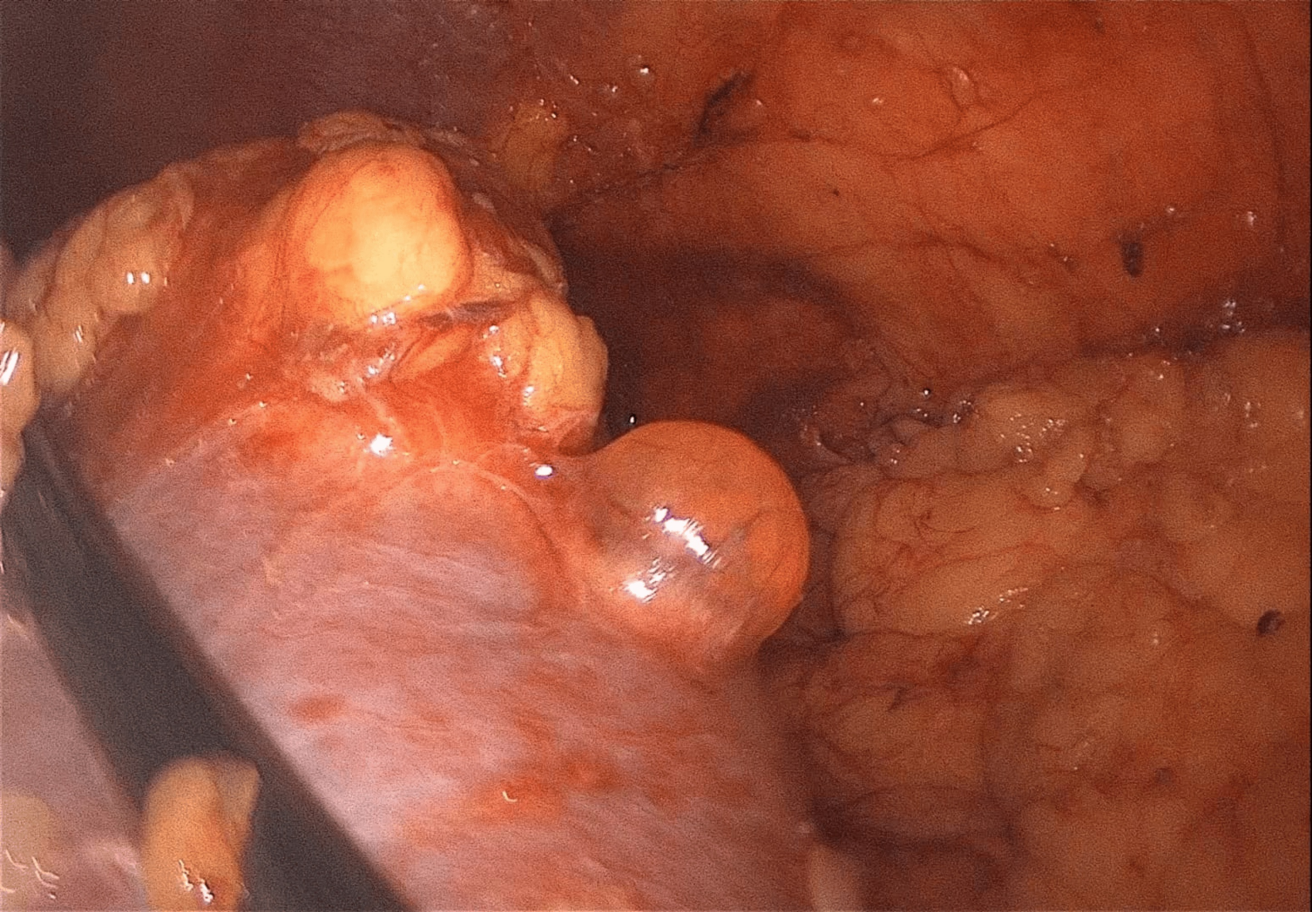
Intraoperative image during inoculation of the tumor from the base

**Figure 5:**
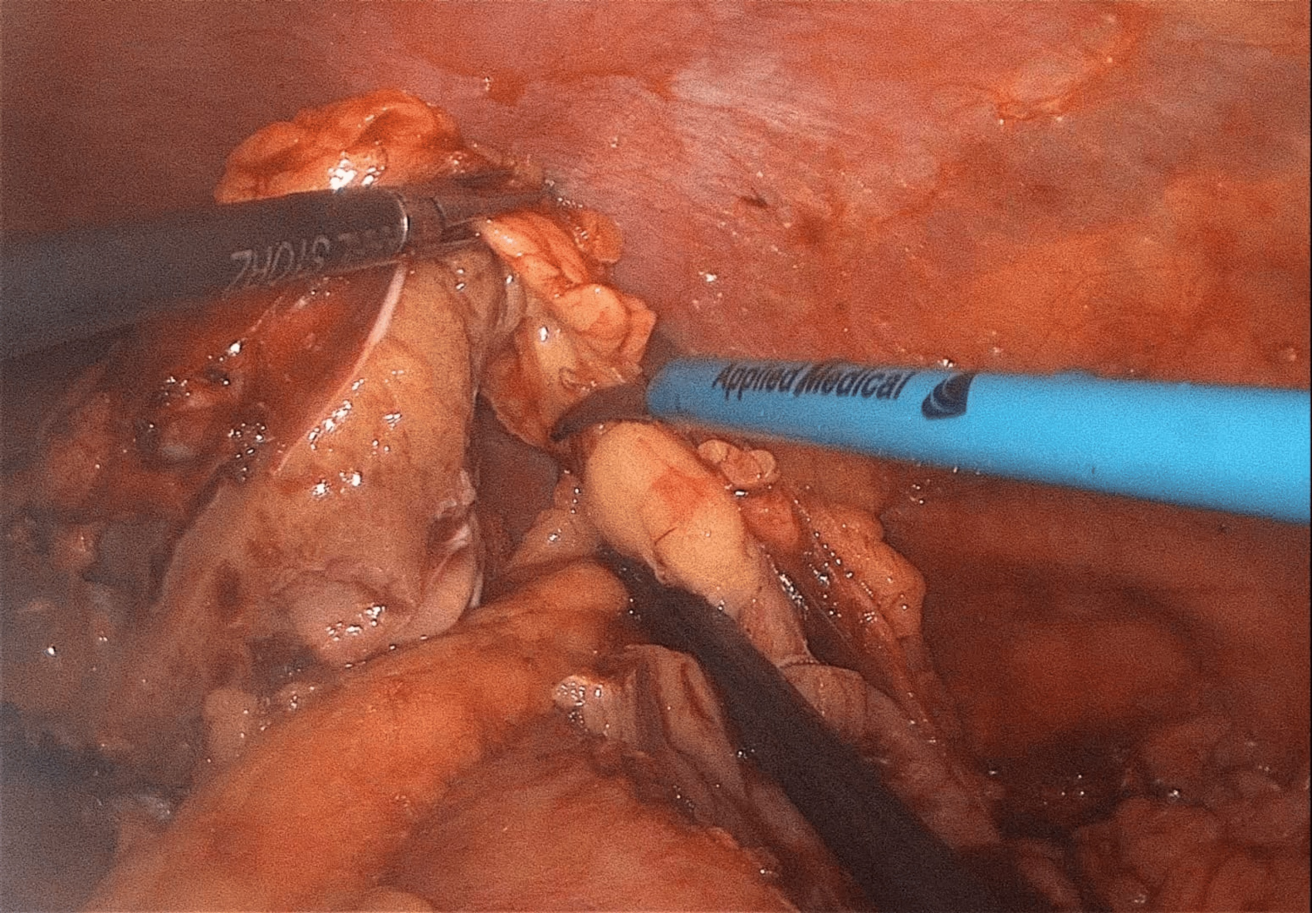
Intraoperative image during inoculation of the tumor from the base

**Figure 6:**
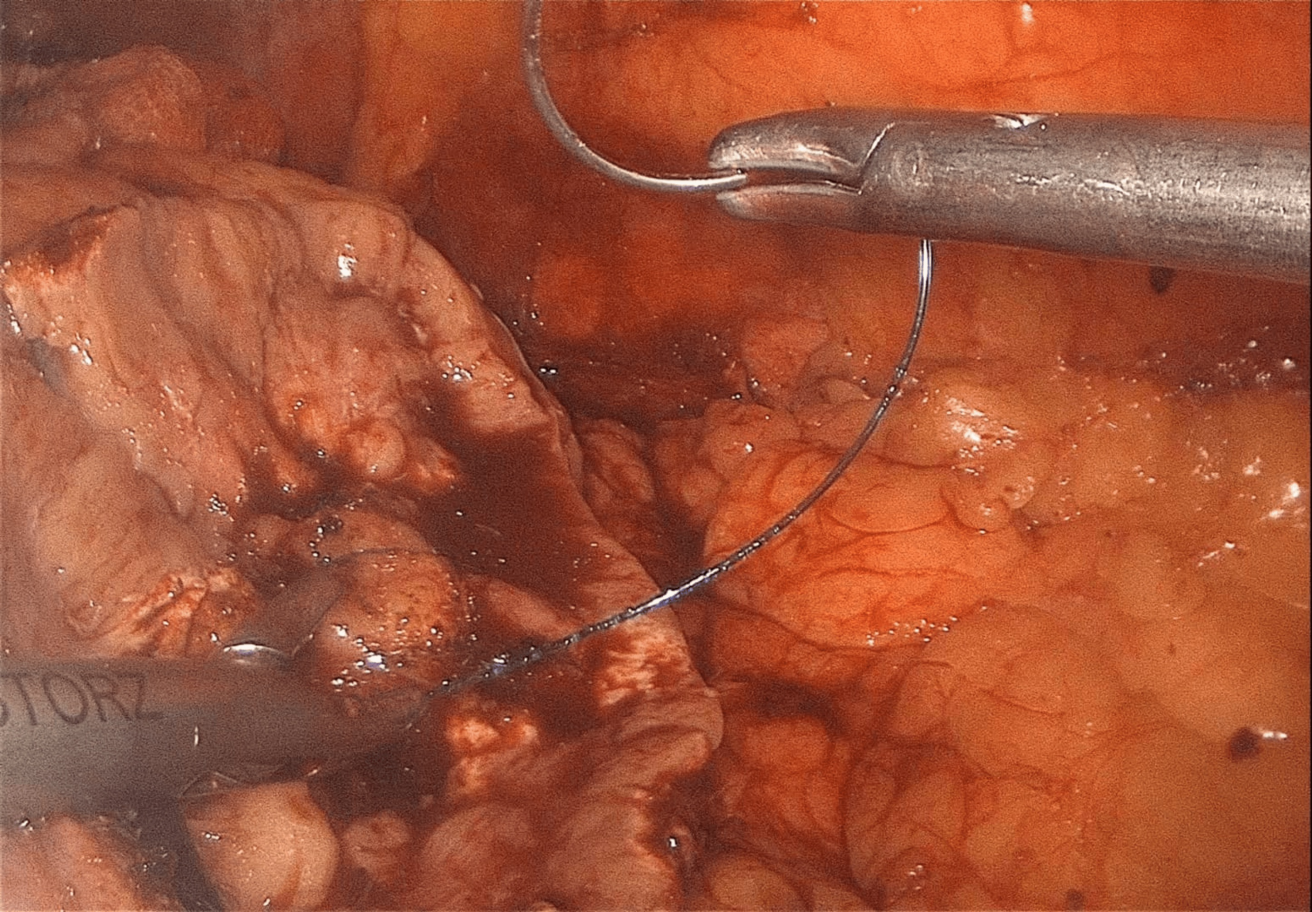
Intraoperative finding of hemostatic suturing during laparoscopic partial nephrectomy

The patient was managed successfully with laparoscopic partial nephrectomy using an inoculation technique with a warm ischemia time of 18 minutes (Figures 4-5) and had an uneventful postoperative course (Figure 6).

The histopathology result revealed three multifocal masses, the largest being 4 cm in its greatest dimension, representing clear cell RCC. The histopathologic grade showed grade G3 with tumor necrosis features. The tumors were limited to the kidney parenchyma with negative margins. There were no lymphovascular and no features of sarcomatoid and rhabdoid tumors.

**Figure 7:**
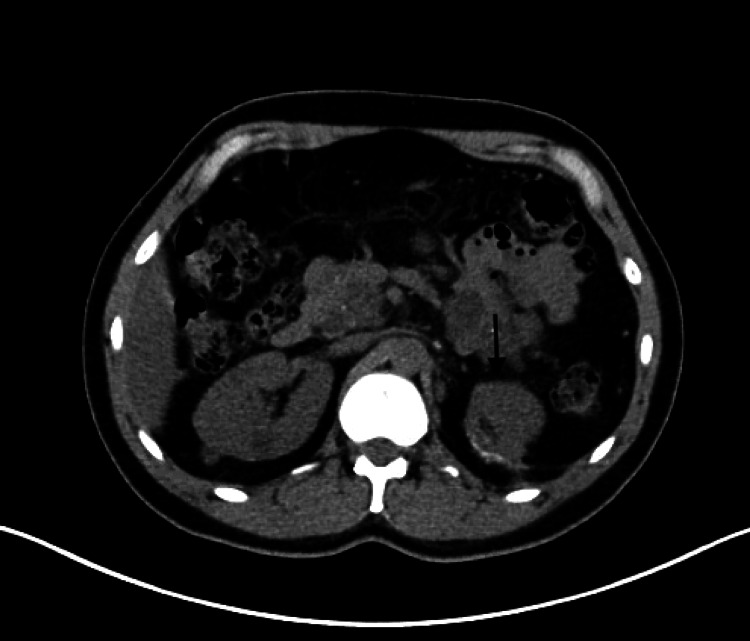
CT abdomen (three months post partial nephrectomy) revealed no tumor recurrence CT: computed tomography

The patient tolerated the procedure well and had an uneventful postoperative course. The patient was followed up with a serial CT scan at three months and six months post-op, along with serial creatinine levels at each visit. No remarkable findings were found (Figure 7).

The patient was also referred to ophthalmology, neurology, and general surgery for post-operative consultations and evaluation of his CT findings. No intervention was performed by the consulted specialties.

## Discussion

Through an exploration of the patient's clinical history, imaging results, surgical approach, and postoperative outcomes, this report aims to contribute to the growing body of evidence supporting the effectiveness and feasibility of laparoscopic partial nephrectomy as a preferred surgical intervention for managing multiple complex renal masses in Von Hippel Lindau patients.

VHL is characterized by the development of numerous benign and malignant tumors, along with cysts in other organs. This necessitates an accurate prognosis, expert clinical evaluation, and a timely diagnosis to offer proper therapeutic interventions. VHL disease stands as the oldest recognized, well-defined, and most common hereditary RCC syndrome. Most non-inherited RCC cases have been identified to involve somatic inactivation of the VHL gene [4].

VHL is a rare autosomal dominant hereditary disease characterized by potentially malignant cysts found in various organs. Among these, RCC manifests in VHL in 24% to 45% of individuals with VHL disease [5], displaying a high recurrence rate and typically appearing in young patients with a median age onset of 39 years old. In VHL-affected individuals, the predominant form of RCC is clear cell RCC, commonly diagnosed between 25 and 40 years of age and recognized as one of the leading causes of mortality among VHL patients [6]. Typically, clear cell RCCs measuring under 3 cm often remain asymptomatic for extended periods and carry a low risk of metastasis in VHL patients. However, as the disease progresses, larger renal carcinomas may present with flank pain, hematuria, or the presence of a palpable mass in the flank region, which tends to become bilateral, multiple, and prone to recurrence [6,7].

The diagnosis of renal lesions in VHL patients may raise a challenge, particularly for asymptomatic individuals. MRI is the standard tool for diagnosing and staging renal lesions in VHL disease [4].

It is considered a challenge to manage patients with multifocal renal tumors and hereditary syndromes predisposing to the formation of RCC. Two main goals guide management: first, to avoid metastasis, and second, to delay dialysis as much as possible. Therefore, determining the appropriate intervention timing and approach remains an immense challenge, requiring comprehensive clinical assessment and judgment.

After reviewing the literature, no universally agreed-upon guidelines were identified for managing such cases or similar to ours. In most cases found, neoadjuvant treatment was offered. Although any size of RCC carries metastatic potential, in principle, a "watch and wait" approach has been recommended for lesions smaller than 3-4 cm in diameter due to their low metastatic risk. Numerous studies have demonstrated the association between larger tumor size and increased risk of metastases in patients with VHL disease, leading to a generally recommended threshold of a 3 cm diameter for surgical resection [8,9]. Gupta et al. highlighted the feasibility of excising RCCs larger than 4 cm in patients with VHL disease [10]. Despite the debated cut-off limit for deciding on when to surgically intervene, organ-preserving strategies such as nephron-sparing surgery or partial nephrectomy should be performed whenever technically feasible. Innovative nephron-sparing treatment techniques such as cryotherapy, radiofrequency, or microwave ablation, which require imaging control, are also being utilized [11]. Renal transplantation has been successfully performed in VHL syndrome patients who require bilateral nephrectomy for multiple, advanced-stage RCCs and renal failure. Kidney transplantation is not yet extensively adopted as a treatment option for VHL syndrome due to limited research and concerns regarding post-transplant immunosuppression potentially increasing the risk of tumor recurrence. However, in a study comparing 32 patients with VHL syndrome with renal transplants to 32 patients without demonstrating any significant differences when comparing the survival prognosis or the changes in renal function over a four-year active follow-up period [12].

All these innovative treatment techniques hold significant importance for VHL patients, as they increase the quality of life by delaying the need for renal dialysis and preventing distant metastases [6].

## Conclusions

Complex renal masses can be managed with renal-sparing surgery through thoroughly experienced surgeon skills. We recommend an active multidisciplinary team approach involved in patient follow-up. We also recommend serial radiological examinations to tackle and detect the development and progression of RCC and other VHL syndrome-associated tumors to maintain the greatest possible normal organ function and to prevent distant metastases and fatal disease outcomes. In addition, we recommend early diagnostic measures by performing genetic tests on patients and family members, leading to earlier detection of malignancies.
